# A Case of μ Heavy and λ Light Chain Amyloidosis in a Patient With Bi-Clonal (IgM κ and λ) Gammopathy Treated With Daratumumab

**DOI:** 10.7759/cureus.56994

**Published:** 2024-03-26

**Authors:** Armaan Dhaliwal, Ashish Tripathi, Soumiya Ravi

**Affiliations:** 1 Internal Medicine, University of Arizona College of Medicine, Tucson, USA; 2 Internal Medicine, Dayanand Medical College and Hospital, Ludhiana, IND

**Keywords:** monoclonal antibody, lymphoplasmacytic lymphoma, heavy chain, amyloidosis, daratumumab

## Abstract

Our case report is of an elderly male with a history of IgM κ lymphoplasmacytic lymphoma (LPL) presenting with generalized neuropathy and weakness. Due to his LPL history and worsening renal function, he underwent a renal biopsy revealing the presence of μ heavy and λ light chains, revealing a diagnosis of amyloidosis with unbound heavy & light chains (AHL), a rare type of amyloidosis. His bone marrow biopsy demonstrated κ light chain restriction by flow cytometry and amyloid deposition. The patient’s serum had elevated free κ and λ light chains with a free light chain (FLC) ratio of 3.17. Serum immunofixation was positive for IgM κ and λ light chain clones. He completed six cycles of cyclophosphamide, bortezomib, dexamethasone, and rituximab (CyBorD+R), normalizing the FLC ratio. Still, he continued to present with persistently elevated M protein, IgM κ, and λ light chains on immunofixation. Thereafter, daratumumab, a human monoclonal antibody directed against CD38 expressed on myeloma cells was initiated, which led to a negative immunofixation study after two cycles accompanied by a reduction in protein excretion in the urine. The patient achieved a complete hematological response with daratumumab. To date, our case is the only reported μ heavy and λ light chain amyloidosis patient with bi-clonal (IgM κ and λ) gammopathy to be successfully treated with daratumumab.

## Introduction

Amyloidosis encompasses a diverse group of protein-folding disorders resulting in the deposition of misfolded proteins in different body organs. The most widely known subtype is light chain (AL) amyloidosis, characterized by the excessive production of light immunoglobulin chains, namely κ and λ. Immunoglobulin (Ig)M monoclonal gammopathy accounts for 5-7% of AL amyloidosis cases [1]. Lymphoplasmacytic lymphoma (LPL) causes AL amyloidosis in about 3% of the cases [2].

Amyloidosis with heavy chains (AH) and amyloidosis with unbound heavy and light chains (AHL) are uncommon forms of immunoglobulin-derived amyloidosis. Three types of AH/AHL have been described based on the immunoglobulin class of the heavy chain involved - alpha, gamma (most common), and μ (least common) [3].

Both light and heavy chain subtypes usually affect the elderly and have an underlying plasma cell/B-cell lymphoproliferative disorder [4]. Kidneys are commonly involved in AL, AH, and AHL cases with proteinuria with or without renal insufficiency as the presentation. Renal AH/AHL cases have been found to have a lower incidence of concurrent cardiac involvement and a higher likelihood of presenting with circulating intact monoclonal immunoglobulin [3].

Here, we present a case of AHL amyloidosis (μ heavy chain and λ light chain) in a patient with a known diagnosis of lymphoplasmacytic lymphoma. The patient was successfully treated with daratumumab, an anti-CD38 monoclonal antibody.

## Case presentation

A 68-year-old male with a past medical history of chronic kidney disease presented to his family physician's office in 2015 for a routine checkup. His primary care physician (PCP) ordered routine blood work revealing paraproteinemia, prompting further testing and he was found to have IgM κ monoclonal gammopathy. Thereafter, a bone marrow biopsy was performed at the time which showed scattered plasma cells on CD138 stain (<5%), with flow cytometry showing 13% of the cells to be of the κ-restricted monoclonal B-cell population. He was diagnosed with lymphoplasmacytic lymphoma (LPL) and he did not require any treatment at that time except close monitoring.

In 2021, he complained of persistent fatigue, generalized numbness, and tingling. Due to his history of LPL and worsening renal function found on blood work, he underwent a left kidney biopsy, which showed AHL amyloidosis with μ heavy chain and λ light chain type on laser microdissection with tandem mass spectrometry (LMD/MS), a highly sensitive and specific test to type amyloidosis. His echocardiogram revealed mild to moderate concentric left ventricle hypertrophy, indicating cardiac involvement of amyloidosis. CT scan of the chest/abdomen/pelvis showed a slightly prominent liver (18.1 cm) with fatty infiltration. A nerve conduction/electromyography (NCS/EMG) study for his neuropathy revealed no significant evidence for a demyelinating neuropathy. Eventually, he was seen in the hematology clinic for this new AHL diagnosis with new end organ damage.

**Figure 1:**
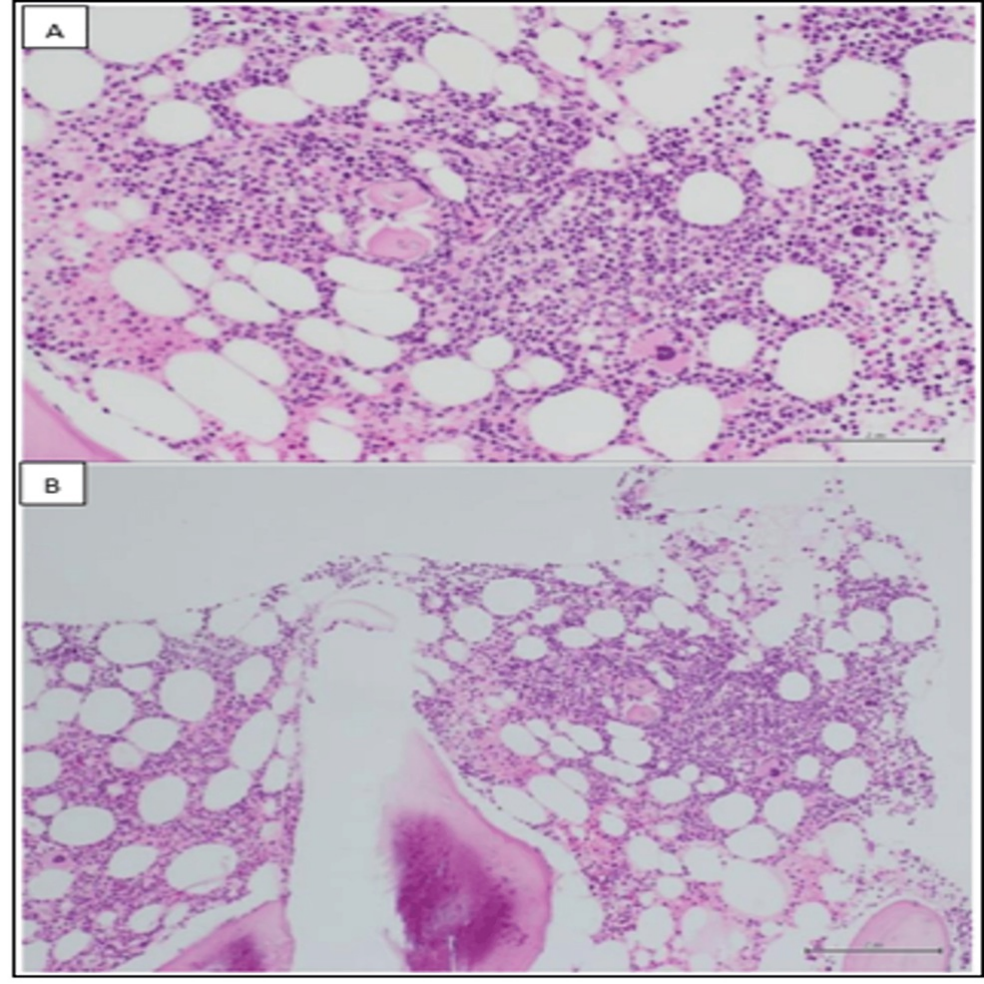
Immunohistochemical study results (I) A and B panels demonstrate a lymphoid aggregate with small lymphocytes and few plasma cells within the bone marrow core biopsy.

**Figure 2:**
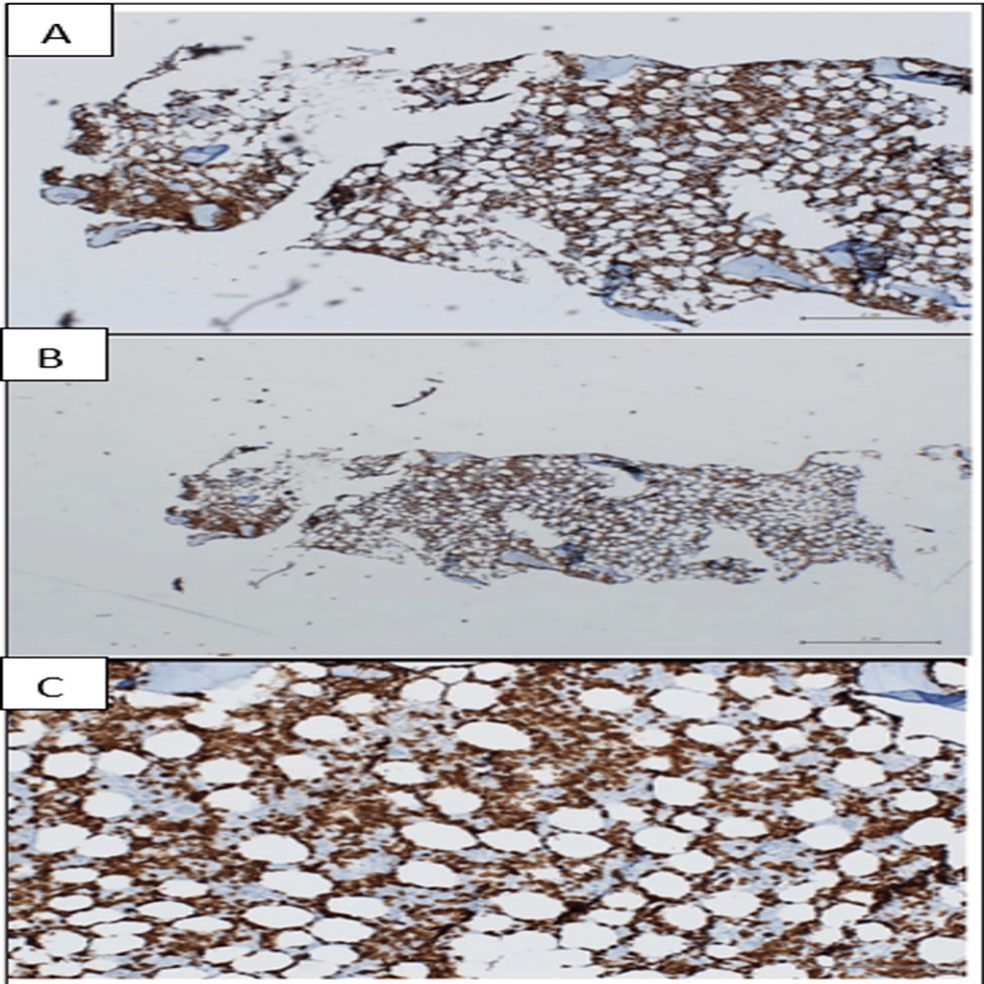
Immunohistochemical study results (II) The three panels A, B, and C depict CD20 staining, highlighting a diffuse interstitial and focally nodular CD20+ lymphocyte infiltrate within the bone marrow.

Repeat bone marrow (BM) biopsy showed that the marrow was normocellular (up to 50%) and contained a normal number of plasma cells that appeared polyclonal by in situ hybridization. B-cells were increased, as shown by an immunohistochemistry (IHC) study (30% of marrow cellularity) and it showed κ light chain restriction by flow cytometry, as shown in Figures 1-2.

**Figure 3:**
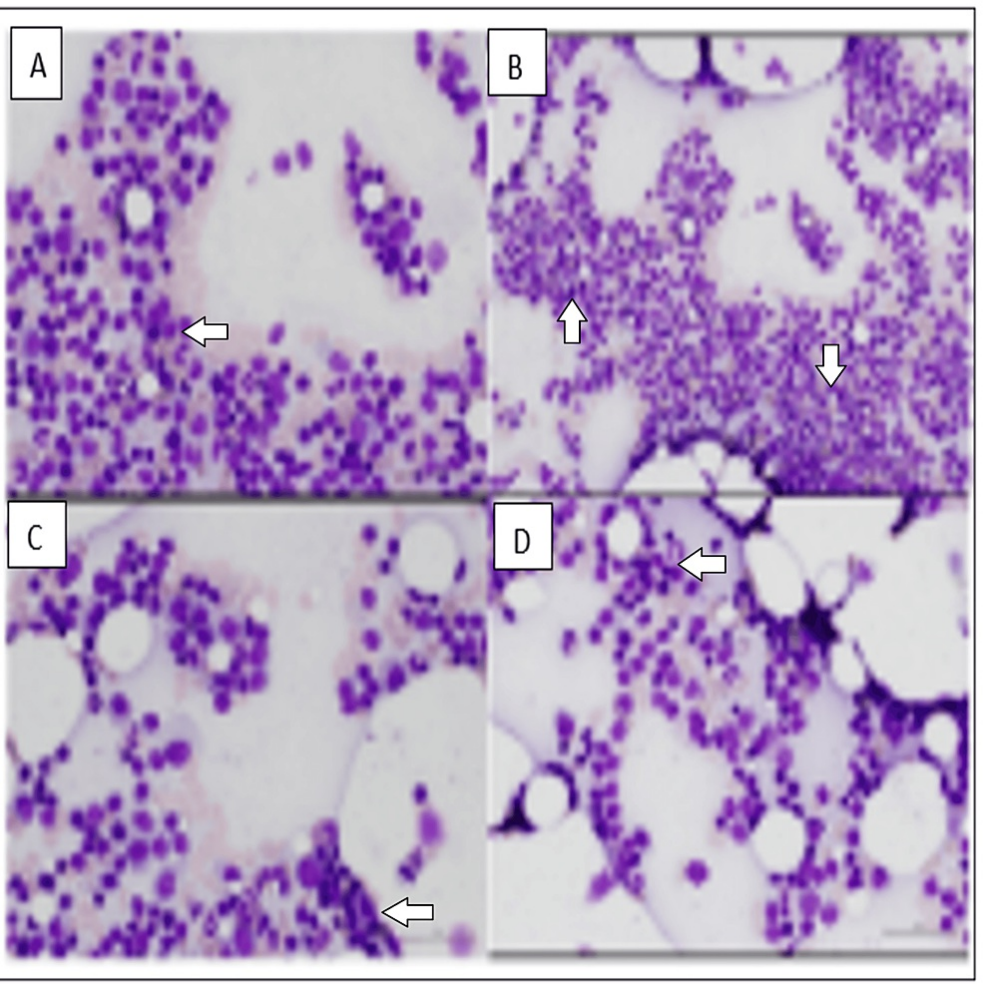
Immunohistochemical study results (III) Multiple panels A, B, C, and D show focal areas of bone marrow aspirate with increased small mature lymphocytes and plasmacytoid lymphocytes.

The aspirate differential showed focal increases in small and plasmacytoid lymphocytes, consistent with the patient’s history of lymphoplasmacytic lymphoma, as represented by Figure 3.

**Figure 4:**
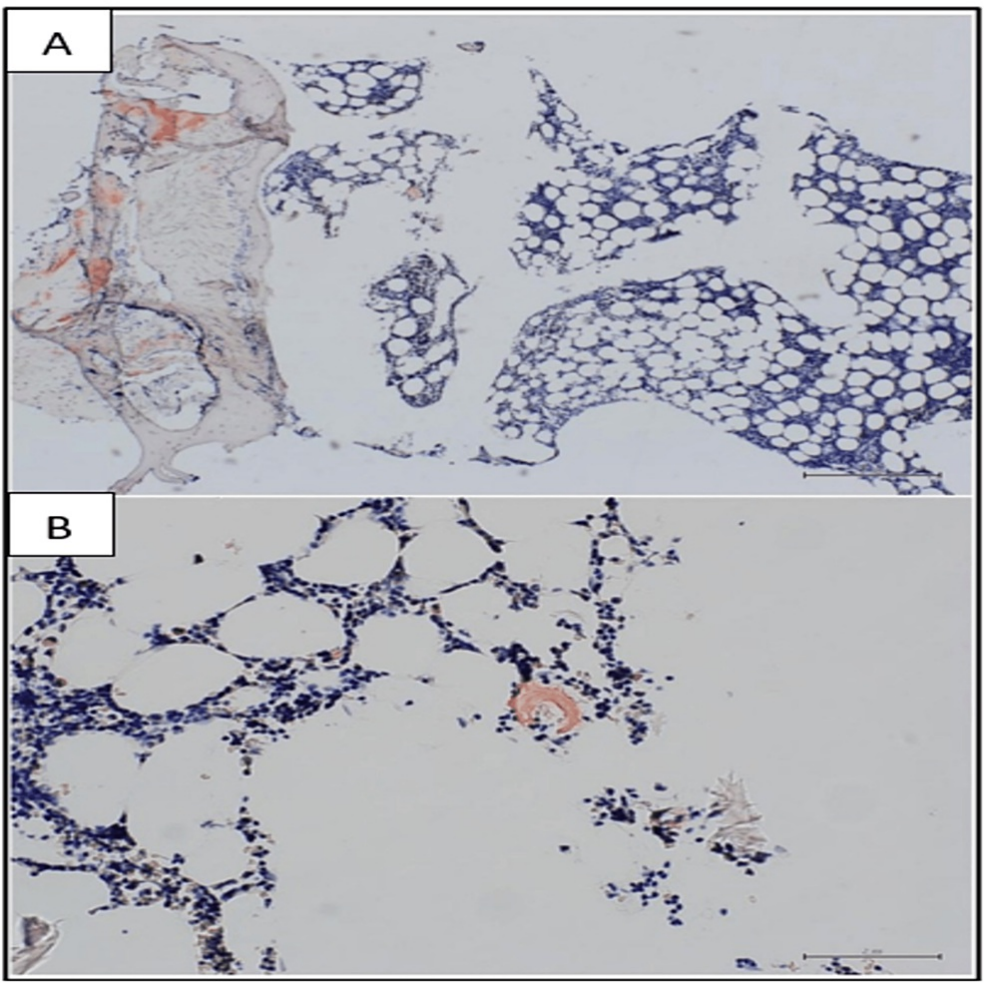
Immunohistochemical study results (IV) The A panel depicts a light microscopy photograph with a Congo red stain showing amyloid deposition in the bone marrow. The B panel is the darkfield photograph with a Texas red fluorescent filter accentuating the congo red staining of amyloid.

Amyloid was detected by Congo red stain as depicted in Figure 4.

There was no evidence of a λ restricted plasma cell or lymphocyte population to explain the finding of amyloidosis (λ +) in the kidney. Liquid chromatography-tandem mass spectrometry (LCMS/MS) was performed on peptides extracted from Congo red-positive areas of the specimen, which detected a peptide profile consistent with ALH (λ light chain and μ heavy chain) type amyloid deposition. The free κ light chain and free λ light chain were 91.67 mg/L and 28.94 mg/L, respectively, with a free light chain (FLC) of 3.17. Repeat serum immunofixation showed two different monoclonal clones, an IgM κ clone, and a λ light chain clone.

The patient was finally diagnosed with AHL amyloidosis with renal, cardiac, and skin involvement. The difference between involved and uninvolved light chains was 62.73 mg/L; N-terminal prohormone of brain natriuretic peptide (NT-proBNP) was high at 1,144 pg/mL with high sensitivity (HS)-Troponin level of 35 ng/L. It was classified as stage 1 based on the Mato 2012 amyloidosis staging.

The patient was initially started on a combination of bortezomib, rituximab, and dexamethasone to treat the underlying clone of LPL. He was also started on acyclovir for antiviral prophylaxis. He had a dFLC (difference between involved and uninvolved LCs) of 62.73 mg/L. Due to a suboptimal M protein reduction, cyclophosphamide was added to the original combination. After a total of six cycles of chemotherapy, the patient achieved a very good partial response with a dFLC of 3.16 mg/L. His κ /λ ratio normalized with the disappearance of κ LCs from the urine, but his serum electrophoresis continued to be positive for IgM κ and λ LCs. His M spike and IgM levels remained slightly elevated at 0.2g/dL and 340mg/dL but stable without complete normalization. He was eventually started on subcutaneous daratumumab 1,800 mg for eight weekly doses initially, comprising cycles 1 & 2, with each cycle's duration being four weeks. For cycles three to six, he received daratumumab 1,800 mg once every two weeks for a total of another eight doses.

**Figure 5:**
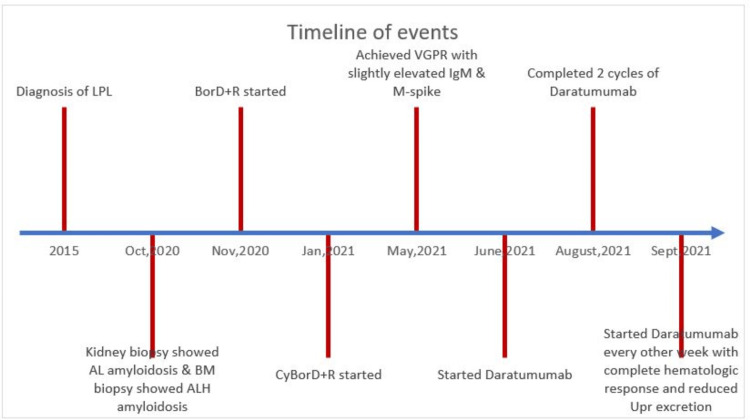
Timeline of events of the case LPL- lymphoplasmacytic lymphoma, AL- light chain amyloidosis, BM- bone marrow, ALH- heavy and light chain amyloidosis, CyBorD+R- cyclophosphamide, bortezomib, dexamethasone and rituximab, VGPR- very good partial response, Upr- urine protein

The patient continued to have a very good partial response (VGPR) response to daratumumab with reduced dFLC of 2.2 mg/L and normalized κ /λ ratio. Serum immunofixation electrophoresis before daratumumab initiation was positive for monoclonal gammopathy, IgM κ, and λ types. Immunofixation became negative after two cycles of daratumumab, and the patient is now in complete hematological response. Urine electrophoresis was negative for monoclonal light chains both before and after therapy. His cardiac biomarkers continue to stay elevated with a decrease in urine protein excretion from 3491 mg/day to 2488 mg/day but still short of <30% decrease in urine protein (UPr) excretion to be considered an adequate renal response. The patient reports improvement in fatigue and denies the development of any adverse effects to the therapy. The clinical course is depicted in Figure 5.

## Discussion

AL amyloidosis usually involves the deposition of light chains like κ and λ with the deposition of heavy chains, namely gamma, alpha, and μ observed in a minority of cases. IgM-related amyloidosis, including LPL, usually involves the deposition of light chains. In a multicentric French study of 72 patients with IgM-related amyloidosis, none had AH/AHL amyloidosis reinforcing the rarity of the disease [5]. μ heavy chain amyloidosis is the least common form of heavy chain amyloidosis. μ heavy chain diseases (μ -HCDs) display clinical heterogeneity and present as varied courses with only six cases of μ-HCDs described in the literature that were discovered to have amyloidosis described in Table 1 [6-11].

**Table 1:** . Depiction of the seven cases of heavy chain amyloidosis including the presented case based on literature review. IE- immunoelectrophoresis, BM- bone marrow, LP- lymphoproliferative, CLL- chronic lymphocytic leukemia, MM- multiple myeloma, MGUS- monoclonal gammopathy of undetermined significance, WM- waldenstrom macroglobulinemia, CyBorD+R- cyclophosphamide, bortezomib, dexamethasone and rituximab

Age	Serum IE	Urine IE	BM biopsy	Organs affected	Underlying LP disease	Treatment	Site of μ chain detection
Gender							
58 M [6]	Κ & μ	Κ	-	Carpal tunnel, lymph nodes, liver, spleen	CLL	Chlorambucil	Serum
						Steroids	
52 M [7]	Κ & μ	Κ	Κ & μ	Joints, carpal tunnel, lymph nodes, liver	MM	Cyclophosphamide	Serum & bone marrow
						Phenylalanine mustard,	
						prednisone	
56 M [8]	λ	λ	Not done	kidneys	BM biopsy not done	None	Kidney
41 M [9]	μ	Κ & μ	-	Tongue, carpal tunnel	CLL	Melphalan	Serum & urine
						Prednisone	
						Dimethyl sulfoxide	
61 F [10]	Κ	-	-	Kidneys	MGUS	Cyclophosphamide	Kidney
						Bortezomib	
						Dexamethasone	
75 M [11]	Κ	-	-	Kidneys	MGUS	Cyclophosphamide	Kidney
						Prednisolone	
74 M (present)	Κ & λ	Κ	λ & μ	Heart, liver, nerves, kidney	WM	CyBorD+R	Bone marrow
						Daratumumab	

Based on the literature review, none of the LPL-related amyloidosis cases had μ heavy chain amyloid fibrils. In addition to the presence of μ heavy chains, this case was found to have biclonal light chain (κ & λ ) gammopathy.

Rituximab-based regimens are the mainstay therapies for amyloidosis, including those related to IgM disorders. CyBorD+R is a commonly used combination that was also the primary mode of therapy employed in the discussed case. This regimen combines four medications that induce apoptosis and lead to tumor cell destruction; cyclophosphamide is an alkylating agent, bortezomib is a proteosomal inhibitor, dexamethasone in high doses can kill myeloma cells, and rituximab is a monoclonal antibody directed against CD20 on B-lymphocytes. CyBorD+R was eventually replaced with subcutaneous daratumumab, which destroys plasma cells by binding to CD38. CyBorD+R achieved a very good partial response (VGPR) to the chemotherapy but with persistent slight elevation of IgM and M-spike. Daratumumab has been successfully added to the existing regimen of CyBorD/CyBorD+R for the management of AL amyloidosis after the CD38 targeting antibody showed remarkable hematologic and organ responses in the Andromeda study [12]. A clinical trial NCT04131309 is currently underway to assess the efficacy of a single agent subcutaneous daratumumab in newly diagnosed AL Amyloidosis patients.

The six cases of μ heavy chain amyloidosis were treated with combinations, not including daratumumab. The presented case is the first case of μ heavy chain amyloidosis with biclonal light chain (κ & λ) gammopathy successfully achieving a complete hematologic response with subcutaneous daratumumab formulation. The patient has successfully completed two cycles of daratumumab 1,800 mg i.e, eight weekly doses without any adverse effects or disease progression, with daratumumab 1,800 mg injections switched to twice a month now for another four months and eight more doses of therapy.

A report of a transplant-ineligible AHL patient with gamma heavy chains achieving complete hematological response after eight doses of daratumumab and complete cardiac and renal response after 30 doses of daratumumab was published, reinforcing the belief that daratumumab can be an appropriate therapy for amyloidosis with heavy chains [13]. To date, our case is the only reported publication of an AHL patient with μ heavy chains and λ light chains with biclonal (IgM κ & IgM λ) gammopathy, achieving and sustaining a complete hematologic response to daratumumab after two cycles. The patient still has not shown a cardiac response, as suggested by the persistent elevation of NT-proBNP and HS-troponin. Renal response characterized by the decrease in Upr excretion is encouraging, with further decrease in Upr excretion expected with subsequent daratumumab doses.

## Conclusions

This case report highlights a case of LPL with the concomitant presence of κ and λ LCs along with μ heavy chains, a rare amyloidosis presentation. There are reports of amyloidosis with μ heavy chains in the literature, but none associated with LPL. These patients were treated with agents other than daratumumab, given the rarity of AH/AHL cases and the fact that daratumumab is a novel agent. Our LPL patient with μ heavy chain AHL amyloidosis reached a response limit to CyBorD+R, requiring management with single agent daratumumab. Daratumumab is now used as front-line therapy for amyloidosis, the primary light chain subtype. AHL/AH and especially μ heavy chain amyloidosis have no diagnostic, treatment, or monitoring guidelines to date. It is intended that the presented case can assist in considering daratumumab for the management of heavy-chain amyloidosis.
